# In reply

**DOI:** 10.1093/oncolo/oyag121

**Published:** 2026-05-08

**Authors:** Marie Dreyer, Al B Benson III

**Affiliations:** Robert H Lurie Comprehensive Cancer Center, Northwestern University, Chicago, IL, United States; Robert H Lurie Comprehensive Cancer Center, Northwestern University, Chicago, IL, United States

We thank the authors of this letter regarding our recent publication, “A phase II study of FOLFOX combined with nab-paclitaxel in the treatment of metastatic or advanced unresectable gastric, gastroesophageal junction adenocarcinoma: a Big Ten Cancer Research Consortium trial”^1^ for the opportunity to discuss the concerns raised and to provide additional context.

First, we would like to clarify the study timeline. Although the enrollment period spanned from 2017 to 2023, the majority of patients were accrued prior to the widespread adoption of immunotherapy in the first-line treatment of metastatic gastric and gastroesophageal junction adenocarcinoma. Importantly, regulatory approvals for the use of PD-1 inhibitors in this setting did not occur until late 2021 and early 2022 in many countries. Our protocol was developed and activated before these changes in standard of care and was not designed to select for or against PD-L1 expression. As the field rapidly evolves, we agree that PD-L1 status and access to immunotherapy are important considerations for future study designs. In this context, our FOLFOX-A regimen (with nab-paclitaxel) may potentially provide a more potent backbone with an improved hematologic toxicity profile.

Regarding the concern about the FOLFOX backbone, it is accurate that our regimen omitted the 5-fluorouracil (5-FU) bolus. However, it is important to note that the bolus 5-FU is often excluded in both routine clinical practice and various clinical trials due to its toxicity and uncertain contribution to efficacy. In colorectal cancer and other gastrointestinal cancers, the bolus is only used in the curative intent adjuvant setting and is omitted in routine metastatic settings, with some even advocating for its exclusion altogether.^2^^,^^3^ The superior outcomes demonstrated compared to FOLFOX are not attributable to the omission of the bolus in the FOLFOX-A regimen. Notably, the FLOT regimen—referenced in the correspondence and widely used in the perioperative setting—also does not include a 5-FU bolus, supporting the notion that this component may not be critical for cytotoxic synergy in gastric cancer.

Lastly, while we acknowledge that FLOT is primarily utilized in the perioperative treatment of locally advanced disease, several studies have evaluated FLOT in the metastatic setting as well.^4–7^ Our reference to FLOT was made in the context of relative activity and toxicity comparisons. Our regimen of FOLFOX-A demonstrated similar efficacy with what appears to be a more manageable toxicity profile, meriting further exploration in select patient populations.

We again thank the authors for their comments and share their commitment to improving outcomes for patients with metastatic gastric cancer, including those who may not be candidates for immunotherapy. Continued investigation into effective, tolerable non-immunotherapy combinations remains an important priority.

## Author contributions

Marie Dreyer (Writing—original draft, Writing—review & editing) and Al B. Benson (Writing—original draft, Writing—review & editing)

## Funding

No funding.

## Conflicts of interest

A.B.B.: BMS, Astellas, Apexigen, PrecisCA, Array (Pfizer) Novartis DMC, Amgen, Terumo, Mirati, GSK, Bayer, Aveo DMC, Gusto, Boehringer Ingelheim, Abbvie, Artemida, White Swan, Harborside Press, Patient Resources, LLC, Axis Medical Education, Envision Communications, Trialcard Incorp, Tempus Labs, Inc, Aptitude Health, Therabionic, Clarivate Analytics US LLC, Tukysa, Natera, Janssen, AIM Immuno Tech, Nuvation Bio, Xencor, Janssen, Xenor, Boston Scientific. The other authors indicated no financial relationships.
